# Exploring caregivers’ perceptions of community-based service requirements of patients with spinal cord injury: a qualitative study

**DOI:** 10.1186/s12875-023-02051-3

**Published:** 2023-04-11

**Authors:** Nasrin Galehdar, Heshmatolah Heydari

**Affiliations:** 1 Social Determinants of Health Research Center, School of Allied Medical Sciences, Lorestan University of Medical Sciences, Khorramabad, Iran; 2 Social Determinants of Health Research Center, School of Nursing and Midwifery, Lorestan University of Medical Sciences, Khorramabad, Iran; 3 French Institute of Research and High Education (IFRES-INT), Paris, France

**Keywords:** Spinal cord injury, Community-based services, Caregivers, Qualitative research

## Abstract

**Background:**

The incidence of spinal cord injury is increasing worldwide. Patients with spinal cord injury and their families face many difficulties during the disease course. Caregivers are more involved with these patients than anyone else, so recognizing patients’ care requirements based on caregivers’ opinions can facilitate care provision to these people. The purpose of this study was to explore caregivers’ perceptions of the community-based services requirements of patients with spinal cord injury.

**Methods:**

This qualitative research was conducted in Iran from Apr 2021 to Dec 2022 using the conventional content analysis method. The participants in the study included family caregivers and providers of home care services to patients with spinal cord injury, who were selected by purposeful sampling. Data were collected by conducting 14 face-to-face interviews and analyzed based on the method proposed by Lundman and Graneheim.

**Results:**

Data analysis led to the extraction of 815 primary codes, which were organized into two themes: community reintegration (with two categories, including the need to provide a suitable social platform and lifelong care) and palliative care (with two categories, including family conference and survival management).

**Conclusion:**

Social facilities and infrastructure should be modified in a way that patients with spinal cord injury can appropriately benefit from community-based care services and an independent satisfactory life. Palliative care should be continuously provided from the time of lesion development until the patient’s death.

## Background

According the report of the global burden of disease study, the prevalence and incidence of spinal cord injury (SCI) are increasing, with a reported prevalence of 20.6 million and an annual incidence of 0.9 million. Among the most important causes of this event are falls and road accidents [1].

The incidence of traumatic SCI in Iran is 10.5 per million; its prevalence is 3 per 100,000, and its mortality rate has been reported as 3.9% [2]. The relatively high incidence of this debilitating condition among youth leads to enormous financial and physical losses. Most of those affected are men in the age range of 19 to 34 years old [3].

Spinal cord injury causes dysfunction in various body systems such as the sensory, motor, musculoskeletal, digestive, urinary, cutaneous, and respiratory systems, as well as psychological disorders [4, 5]. These consequences impose a great work burden on the family and the health system [6] and require spending high costs for managing the patient [7]. During the acute phase, the patient may become completely or partially dependent on others, requiring receiving care for years [6].

Management of chronic diseases, such as SCI, is one of the main challenges of the health system, and expansion of community-based services is a leading strategy to reduce the incidence of hospital-related complications and the duration of hospital stay [8]. Community-based services can have many benefits for patients and health systems, such as reducing the rate of readmission to the hospital and emergency department, upgrading quality of life, and effectively managing limited health resources [9].

After the acute period of the disease, community-based services are necessary for many SCI patients during the recovery period and after acquiring partial recovery. If the patient fails to receive appropriate out-of-hospital care, he/she may need re-hospitalization [10]. The positive consequences of using home care services include a reduction in financial costs and need for hospital-based care and an increase in social participation and patient satisfaction, highlighting the unique role of home care services in today’s world. Besides, technological advances in medicine have created suitable grounds for the expansion of home care provision [11, 12].

The physical complications pertaining to SCI are notably prevalent worldwide. According to the results of a study, 95.8% of SCI patients experienced at least one or more physical problems such as pain, spasticity, sexual dysfunction, and respiratory problems secondary to their injury [13]. Also, these patients may experience psychosocial problems more commonly than the general population [14].

Families and caregivers face many issues and problems during caring for SCI patients at home [15]. Due to various reasons such as the lack of educational, supervisory, and specialized systems, the management of patients with chronic diseases should inevitably pass from the in-hospital phase to home-based and in-society maintenance therapy, as one of the important pillars of the healthcare chain [16].

Every country, based on the context of its health system, as well as political, social, economic, and cultural parameters, employs unique protocols for providing community-based services [17]. Thus, understanding the perception of the individuals involved in care provision, such as family members, patients, caregivers, and professional health service providers, can help identify patients’ care needs. Thus, the purpose of this study was to explore caregivers’ perceptions of the community-based service requirements of patients with SCI.

## Methods

This qualitative study was conducted in Iran from Apr 2021 to Dec 2022 using the conventional content analysis approach.

### Participants

In this study, participants were selected using the purposeful sampling method among key informant and experienced individuals such as family caregivers and formal caregivers involved in providing care to patients with SCI. We tried to recruit participants in a way to have maximal diversity in terms of age, sex, experience, and work duration. Inclusion criteria for health care workers included being engaged in providing community-based services to SCI patients for at least one year, willingness to participate in the study, and ability to take part in an interview. Inclusion criteria for family caregivers and SCI patients were a definitive diagnosis of SCI by a specialist, willingness to participate in the study, and ability to be interviewed.

### Data collection

In this study, data were collected by conducting 14 face-to-face semi-structured interviews. The researcher referred to comprehensive health centers for SCI patients to recruit participants. In these centers, interviews were initially conducted with formal caregivers. Based on the data gathered in this step, sample recruitment continued in other places. The researcher established a close and direct relationship with the participants in order to acquire authentic and accurate information.

General questions for guiding the interview were: How do you describe your experience of community-based services? What are the challenges of providing home care to patients with SCI? and What are the care needs of these patients in the community? The researcher used probing questions to direct the interview toward the objectives of the research. It should be noted that based on the data obtained during the study, the main question of the study was slightly modified according to the study’s objectives. The duration of each interview was between 15 and 40 min, and an electronic device was used to audio-record the interviews.

### Data analysis

In this study, data analysis was performed simultaneously with conducting the interviews using the method proposed by Lundman and Graneheim [18]. The text was transcribed verbatim immediately after each interview, and the transcribed texts were read several times to extract primary codes. After that, related primary codes were merged together to form categories based on their similarities. Finally, the concepts hidden in the data were extracted.

### Trustworthiness

To ensure the accuracy and rigor of the data, the criteria of credibility, transferability, and confirmability were assessed as proposed by Lincoln and Guba [19]. For this purpose, the researcher established a long-term relationship with the participants to gain their trust. After extracting initial codes, the participants’ opinions were sought about their correctness and interpretations, and in the case of any inconsistency, necessary amendments were made. Also, two university professors who had expertise in the field of qualitative research were requested to express their opinions on the codes selected and their classification. It was also tried to recruit participants with maximal diversity in terms of knowledge, experience, length of service, place of service, age, and gender. Regarding transferability, the study’s protocols in different phases and the participants’ information were described in detail. For confirmability, two researchers independently collected and analyzed the data until reaching a consensus.

### Ethics approval and consent to participate

All methods were performed in accordance with the relevant guidelines and regulations of the declaration of Helsinki (ethical approval and consent to participate). The methods and aims of the study were explained to all participants, and necessary assurance was given to them regarding the confidentiality and anonymity of their information and audio files. Informed consent was obtained from all participations. The participants had the right to withdraw from the study at any time. Ethical approval was obtained from the ethics committee of Lorestan University of Medical Sciences (ethics code of IR.LUMS.REC.1395.192).

## Results

In this study, the data were collected by conducting 14 face-to-face interviews. The participants included five women and nine men (Table 1).

Data analysis led to the emergence of 815 primary codes, which were organized under two themes: community reintegration (with two categories, including the need to provide a suitable social platform and lifelong care) and palliative care (with two categories, including family conference and survival management) (Table 2).

**Table 1:** Participants’ characteristics

Participants code	Sex	Age (years)	Level of education	Work experience (years)	Marital status	Position
1	Female	31	Post-graduate	7	Married	Formal caregiver
2	Male	27	Associate Degree	6	Married	Formal caregiver
3	Male	29	Bachelor	1	Single	Formal caregiver
4	Male	32	Bachelor	10	Married	Formal caregiver
5	Male	38	Post-graduate	10	Married	Formal caregiver
6	Male	32	Post-graduate student	1	Single	Formal caregiver
7	Female	32	Bachelor	6	Single	Formal caregiver
8	Male	30	Bachelor	6	Single	Formal caregiver
9	Male	34	Post-graduate student	6	Married	Formal caregiver
10	Male	48	Bachelor	11	Married	Family caregiver
11	Female	33	Diploma	4	Married	Family caregiver
12	Male	39	Bachelor	5	Married	Family caregiver
13	Female	28	Associate Degree	7	Single	Family caregiver
14	Female	30	Bachelor	8	Married	Formal caregiver

**Table 2:** The categories and sub-categories extracted from the data

Themes	Categories	Sub-Categories
**Community reintegration**	The need to provide a suitable social platform	Paying attention to home care requirements
		Social support
		Adaptation of urban furniture
	Lifelong care	Early care
		Continuous care
		Psychological support
		Physical care
		Documentation of the disease course
Palliative care	Family conference	Preparing the family for the loss of the loved one
		Necessity of delivering family care
	Survival management	Not trying to extend the patient’s lifespan
		Respecting the patient’s right of euthanasia

## Community reintegration

Community reintegration entails an outpatient rehabilitation program that facilitates transition from acute care setting to community-based services. This program also help people with chronic or acute conditions who currently live in the community improve their functioning and access to social welfare resources. This theme included two categories: the need to provide a suitable social platform and lifelong care.

### The need to provide a suitable social platform

In order to provide care to SCI patients at the community level, there is a need to create the necessary social infrastructure so that these patients can benefit from the capacity of their families at home. Patients with SCI are likely to experience dramatic changes in their living conditions and may encounter financial hardships. Besides, these patients, due to their physical inabilities, are unable to benefit from their civil rights like other people in society.

Under this category, there were three sub-categories including: paying attention to home care requirements, social support, and adaptation of urban furniture.

#### Paying attention to home care requirements

Data analysis showed that SCI patients could receive appropriate care at their homes. People usually have a sense of belonging to their homes, so receiving home care from family members in a place suitably embellished for taking care of a SCI patient can improve the quality of the services provided.

Data analysis showed that family caregivers delivered their services with love, and this can improve the quality of the services. One of the participants, a family caregiver of a patient with SCI, mentioned: “…my father was unable to move, when he was hungry, he would ask us to feed him…and we would respond eagerly…not because we had to… but because we loved him…“[5]. The same participant highlighted the need for family members to take care of such patients: “…A nurse eventually gets tired and may not dedicate enough time for psychologically supporting the patient, causing him/her to deliver inadequate support, so caregivers should be chosen and hired from family members, such as wives…” [5].

#### Social support

Data analysis showed that SCI patients encountered financial problems and required financial support. Some of the participants were breadwinners of the family, so losing their jobs and income due to their physical problems caused the families financial problems. On the other hand, some of the patients had no health insurance coverage, causing the family to dive deep into a financial crisis due to the additional costs caused by SCI. The participants acknowledged that they could not afford some drugs and equipment, requesting these costs to be covered by governmental organizations. Some families could not even meet their patients’ nutritional needs due to these financial problems, delivering the patient vulnerable and emotionally unsatisfied with the condition. Accordingly, one of the participants stated: “…people suffering from SCI need financial support as their families are economically withered…” [1]. Another participant complained of costly drugs: “… the costs of hospitalization and medications are very high… and much of these costs should be covered by the government…” [5]. Another participant addressed the mental and psychological impacts of financial problems on patients: “….Another problem is therapeutic costs…. the patient feels ashamed when he/she cannot afford these costs, leaving him/her devastated seeing that he/she not only is unable to help the family, but also imposes extra costs and sufferings on the family…” [7].

#### Adaptation of urban furniture

Data analysis showed that the living environment of patients should be adjusted to the nature of their disease. The participants stated that SCI patients’ homes were not suitable for these patients (having stairs, difficulty in access to the bathroom and toilet, etc.). Therefore, furniture should be modified in a way to become suitable for these patients. The participants mentioned that the sidewalks, transportation vehicles, buildings, sports equipment in parks, and holy shrines had inappropriate designs for SCI patients. In this regard, one of the participants stated: “…at the homes of some patients, toilets have stairs, it would be great if an additional stairs-free bathroom on a flat surface can be built for them…“[3]. Another participant recalled taking his patient to a park: “…unfortunately, there was no device in the park that a SCI patient could use…” [4]. The participants specified that there should be specially designed parks for patients with SCI. Addressing this issue, one of the caregivers noted: “…they could have at least built a special park for them…” [4]. Another participant stated that furniture had unsuitable design for these patients, “…there is no adaptation in furniture, stairs, …” [5]. Another participant highlighted the necessity of modifying urban infrastructure: “…We should think of SCI patients when designing urban facilities so that they can move more readily…” [9].

### Lifelong care

Patients with SCI may have to live with limitations for years. According to data analysis, health experts should provide continuous care to SCI patients from the first days of developing the injury until after recovery from the acute phase of the illness. These services should be delivered from the day of admission to the hospital, and the patient’s history, problems, and services received should be monitor and documented throughout this period.

This category contained five sub-categories, including early care, continuous care, psychological support, physical care, and documentation of the disease course.

#### Early care

Data analysis showed that the care services aiming to empower patients should be initiated from the first days of injury development so that patients can experience better outcomes. One of the participants addressed the ramifications of the delayed starting of care provision to SCI patients: “…we had a client who was referred to us ten years after the injury, when he developed deformation, deterioration, and osteoporosis along with numerous other acute illnesses, it is very difficult for such a person to return to normal life, and there is not much that one can do…” [4]. The data indicated that patients might gradually lose their spirit for complying with rehabilitation programs. One of the participants noted: “… after years of entangling with their condition, patients would lose their hope for returning to society…” [4]. The participants mentioned that some families were not familiar with the necessary training on how to provide care to the patient. The early starting of care provision by formal caregivers can help family members learn the necessary training, causing the patient to recover faster. One of the participants noted about some undesirable consequences of the late start of family training: “…after years, family members are still unfamiliar with the necessary trainings, types of massages, and even stretching exercises for a patient who had lost his hands and legs completely…. “[6].

#### Continuous care

Data analysis showed that due to the physical and psychological problems faced by these patients, responsible organizations are required to specify special research teams to identify and monitor these complications. One of the participants addressed this issue as: “… all SCI patients, without exception, develop bed sores within a year… a research team is needed to assess these complications…” [4]. In this regard, another participant noted: “… these patients develop gastrointestinal, skin, joint, and bone problems along with many other complications, so a team of experts needs to continuously monitor these patients to prevent and treat such complications… " [6].

#### Psychological support

Data analysis revealed that society might not have a positive attitude toward SCI patients, compelling the patients to stay at home and avoid going out. On the other hand, the patients may use diapers and catheters to control urination and defecation, causing them psychological distress. Regarding this, one of the participants said: “… Actually, whenever we wanted to take him for a diagnostic workup, we had to apply a catheter. It was hard. Then we wanted to use diapers, but he refused, saying “do not put a diaper on me, do not connect the catheter"…. because he believed that these would make him embarrassed…” [5].

Data analysis showed that SCI patients had the fear of being abandoned. The patients admitted that sometimes they tried not to bother their caregivers and family members because of fear of being abandoned. One of the participants stated: “…most of them are afraid of being abandoned, being forgotten. They think that because they cannot participate in household affairs and communicate with their relatives, they are forgotten by them…” [7].

The results showed that SCI patients saw themselves an extra burden on the family. They felt embarrassed because of being dependent on family members and caregivers even for fulfilling some of their personal needs. In this regard, one of the participants noted: “…some patients see themselves an extra burden on the family, and they feel embarrassed for their dependence for cleaning themselves, going to the bathroom, and taking a shower. Imagine a person who used to bathe or go to the bathroom by himself/herself now needs his daughter, his wife, or a home worker for doing such things…” [7]. Another participant stated: “… the first thing that can help a lot is to offer them psychological counseling by a psychologist or a social worker, which can be very helpful for them…” [5].

#### Physical care

Data analysis showed that patients develop various physical problems such as bedsores, gastrointestinal problems, renal problems, and related complications. In this regard, one of the participants stated: “…bed sore is one of the problems of these patients, they also have urinary and fecal incontinence…” [8]. Another participant addressed these patients’ digestive problems: “…. eating food is hard for these patients…they have many gastrointestinal problems…” [9].

#### Documentation of the disease course

One of the necessities of care provision to patients with SCI is to completely record the patient’s information, including his/her current condition and previous diseases. Thus, if a problem occurs during care provision, it can be traceable. One of the participants shared his experience of dealing with a patient who had his hand broken due to osteoporosis: “…I was not told that the patient had osteoporosis and arthritis since he was a 12-year-old and before the occurrence of SCI… we need to open a detailed medical file for such patients in the case of anything happens….he must have told me that he had osteoporosis…” [4]. The same participant underlined the need for paying attention to underlying diseases, including communicable conditions that may endanger the health of caregivers: “…a client of ours had AIDS, the director of our health institute got needle-stick while taking care of him…another client with AIDS received care for a year and half from a caregiver who was unaware of the situation, only then he realized that the patient had AIDS and hepatitis…” [4].

## Palliative care

Data analysis showed that families and patients face many problems during the care provision process. Early after the incident, family members of SCI patients start to dedicate themselves to provide care to the patient with love and passion without any idea of the patient’s future and the coming hardships awaiting them. However, they gradually become exhausted and hopeless. Data analysis indicated that when family members become aware of the patient’s outlook, they may feel hesitant about continuing care provision. This theme contained two categories, including family conference and survival management.

### Family conference

Palliative care providers are suggested to have a meeting with the patient’s family to explain the situation, particularly for incurable diseases, and to boost the family’s awareness and help them make appropriate decisions and become prepared to face bad news. This category included two subcategories: preparing the family for the loss of their loved one and the necessity of providing family care.

#### Preparing the family for the loss of their loved one

Data analysis demonstrated that families should be prepared in advance for the loss of their loved ones who suffer from an incurable disease. In this regard, one of the participants expressed: “… well, the moment when I faced my father’s death, knowing that it was going to happen sooner or later… that I will see his death…. I was preparing myself for that moment long before it happened…, so I had prepared myself for this moment…” [5]. Data analysis also revealed that after the loss of a loved one suffering from SCI, families might start blaming themselves because of feeling guilty for negligence. One of the participants shared his experience: “…for example, when my client died, his sister started to blame herself because of not bathing her mother…most relatives of these patients felt guilty…” [7].

#### Necessity of providing family care

Data analysis highlighted that families should be informed of the pain and suffering awaiting the patient and themselves. Conversing with the members of the treatment team and hearing other patients’ experiences and problems can help families familiarize themselves with their patients’ future troubles. One of the participants described his regrets over his efforts for prolonging his patient’s lifespan: “…we should see and feel what these patients experience, when going to the hospital … we should note how much pain these patients suffer, violation of dignity…the extent of the humiliation they feel…” [5].

### Survival management

The analysis of the data showed that the length of life of patients with incurable illnesses should be managed so that they and their families suffer the least amount of pain and misery. In this category, there were two subcategories: not trying to extend the patient’s lifespan and respecting the patient’s right of euthanasia.

#### Not trying to extend the patient’s lifespan

According to our results, one of the fundamental needs of patients with SCI was their right to receive palliative care. Some participants were seemed to remorse their efforts for prolonging the survival of their patients. One of the participants described this feeling as: “…If I go back to the time when my father was alive suffering many problems, and I was trying to keep him alive because of my feelings and emotions and our father-son affection, I might choose otherwise to prevent him from experiencing all this pain and agony… I would let peaceful death to embrace him, but the fact is that at that moment and because of my feelings, I caused him to suffer for 10 years…” [5].

#### Respecting the patient’s right of euthanasia

The participants admitted that euthanasia could benefit the patient and the family in some cases. Regarding this, one of the participants noted: “…when someone is at the bottom of the line…. suffering constant pain and agony…euthanasia may let him/her to reach peace…” [5]. Another participant recalled the difficult living conditions of patients and their wishing for death: “…absolute depression… many of them wished their death a thousand times a day…” [6]. A caregiver stated about his experience: “… one of my clients once said to me: if you love me, pray for me to die, what’s the point of life if you are on bed, unable to go out, shopping, cooking food for your family, and helping them… it is better to die…” [7]. Another participant addressed the exhaustion of families and their consent for their patient to die: “… families sometimes prefer their patients to die sooner and become relieved of this situation….“ [9]. Another participant stated: “… one of the patients told me to place Aluminium phosphide (the tank pill) on her bedside…” [6].

## Discussion

The findings of this study showed that community-based service requirements for patients with SCI included community reintegration and palliative care. It is important to pay attention to the need for developing a suitable social platform and appropriate infrastructure for lifelong care, family conference, and survival management.

It is essential to maximally recruit the capacity of families in order to provide quality care to patients with SCI. Family caregivers are in touch with SCI patients and feel committed to them more than any other person [20]. In addition to supporting their patients physically and psychologically, family caregivers help patients accomplish their daily activities [21], reducing the care burden imposed on health systems, the need to use professional home care, and the rate of admission to nursing homes [22, 23]. Various studies have delineated that SCI patients tend to seek their services from specialists [24, 25], which can be due to the insufficient knowledge of general practitioners regarding the management of SCI patients [26]. In Switzerland, although general practitioners report up to 94.3% of SCI cases, only 9.4% of these patients are referred to specialists [27]. The policy of the health system should be in a direction that patients with SCI can receive care in the framework of a referral system, starting from family caregivers at home, followed by professional caregivers, including nurses and doctors, and then if they needed second- and third-level care, they should be referred to specialists and subspecialists.

One of the items that should be taken into consideration when providing care to these patients is to modify the living environment to meet their needs. In line with our findings, the results of another study showed that the problems related to public environments, state policies, and transportation were among the most prominent hurdles impeding these patients’ adaptation to their condition [28]. It is suggested to redesign the living place of patients with SCI according to the level and nature of their injury so that these patients can acquire partial independence in performing some of their activities, and their families can take care of them with more convenience.

Another factor that needs to be taken into mind when providing care to SCI patients is the necessity of delivering comprehensive care to these patients. The findings of this study highlighted the importance of the documentation of the disease process and the patient’s health history. In line with our findings, another study reported that proper documentation should be performed when providing home care to patients with chronic diseases [29]. This process will guarantee the access of caregivers to the patient’s health records so that they can deliver better care services. In addition, documented records can be citable in court when a caregiver is accused of medical errors or negligence.

Our results indicated that patients should receive early care in the health centers specially designed for SCI patients, and these care services should continue to be delivered at the community level. In accordance, the results of another study showed that providing early services to SCI patients in specialized care centers could reduce the duration of hospitalization and related complications [30]. Therefore, it is suggested to admit patients with SCI in specialized care units as early as possible and to start rehabilitation measures in the hospital during the acute period of the disease, followed by home care.

Patients with SCI require physical and psychological support. In parallel with the findings of the present study, various studies have shown that SCI patients are vulnerable to physical problems such as pain, muscle spasms, sexual incompetence, respiratory problems, kidney and urinary tract problems, irritable bowel disease, and bedsores [24, 31, 32]. Another study reported a variable prevalence of 26–96% for chronic pain in SCI patients [33]. Among the factors aggravating these physical symptoms are female gender, being a smoker, and traumatic injuries [32].

It has been reported that spinal cord injuries may ensue psychological problems such as emotional shock, depression, fear of future, loss of dignity, lack of self-care, loss of job, failure to continue education, loss of financial support by monetary institutions, and being deprived of social support [34, 35]. Therefore, continuous professional care should be provided to prevent these patients from developing physical and psychological complications.

In line with the findings of this study, other researchers have elucidated that some patients with SCI suffer from financial problems. The costs of therapeutic and rehabilitation programs for these patients are considerably high, leaving many patients in poverty after the incident, so being supported only by charity institutions may be inadequate for these patients [34, 36]. Therefore, it is required that these patients receive social and financial support from governmental and social welfare organizations, as well as charitable foundations. In accordance, the results of another study showed that social organizations and foundations supporting hospitalized patients and other vulnerable groups of society can play a major role in safeguarding the psychological security of these people [37].

The findings of this study highlighted palliative care as one of the essential care needs of patients with SCI. This important issue encompassed the necessity of providing family care, considering the right of euthanasia for the patient, and preparing the family for the loss of their loved one. In order to deliver effective palliative care, the patient’s family should be informed of the prognosis of the disease and incoming problems from the early days of the injury. One of the important factors that can improve the quality of services is to involve the patient’s family in planning the care program, during which they should receive the necessary training, where a mutual relationship should be formed between the patient’s family and the specialist from the time of diagnosis until the patient’s death [16]. In addition to providing comprehensive support to the patient during the disease course, the patient’s family should also become prepared for his/her death so that they can bear the grief easier [38, 39]. Supporting the family should continue up to one month after the patient’s demise [40]. In this regard, the findings of a study showed that the delivery of palliative care and deciding about ending a patient’s life can vary in different countries and based on the age and functional status of patients with traumatic SCI [41]. Such decisions should be made in accordance with the upper-hand laws and cultural values of society. Nevertheless, factors such as the location of injury, type and extent of SCI, and most importantly, diaphragm functionality, can affect decisions about terminating a patient’s life [41].

One of the issues that came under focus in this study was to consider the right of euthanasia for SCI patients. According to the definition by the World Health Organization, one of the goals of palliative care is to bestow patients a peaceful death [42]. It has been mentioned that we should neither hasten nor delay the death of patients in the final phase of their lives so that they can enter the natural process of death [43], a phenomenon that differs from euthanasia or physician-assisted suicide (PAS). According to the International Association for Hospice and Palliative Care (IAHPC), in countries and states where euthanasia and/or PAS are legal, the palliative care team should neither supervise nor implement euthanasia [44].

Many groups oppose legalizing ending life by healthcare workers, presuming it as an immoral task [45, 46]. Euthanasia is illegal in Iran, like many other countries, which is rooted in people’s opinions and beliefs. Therefore, it is necessary to observe this phenomenon from different social, legal, ethical, financial, and political aspects [47]. However, others argue that people suffering from serious chronic diseases should have the right to choose the time and manner of their own death and believe that PAS and euthanasia should be legalized [48, 49]. Regardless of the fact that people with SCI are prone to commit suicide [50] and the increasing rate of euthanasia in some countries [51], decision-making and arguments with regard to this issue are controversial due to ethical conundrums [52], demanding more research. Nonetheless, it should be clarified that death is a personal issue, and the criteria of a good death vary among societies and are subjected to change over time. The care team’s good relationship with the patient and his/her family seems indispensable for achieving a peaceful death [53]. Similarly, Sheng et al. clarified that caregivers should be aware of the patient’s expected life span, prognosis of the disease, anticipated symptoms, outcomes and complications of resuscitation, and the patient’s desires so that they can completely inform the family and help obviate the barriers of a natural peaceful farewell [54]. A good death occurs at home, without pain, stigma, feeling dependent, or experiencing other distressing events, where financial support is provided for basic needs [55].

One of the limitations of this study was the reluctance of formal caregivers to participate because of fear of the disclosure of some obstacles and challenges in their profession that might have caused them to be fired. So, they were assured that their information would remain confidential to encourage them to participate. Also, some patients and their families were reluctant to be interviewed at home due to security concerns and fear of their pensions being discontinued, whose trust was acquired by the intermediary role of formal caregivers.

## Conclusion

It was found in this study that in order to provide community-based health services to SCI patients, we need to modify social infrastructure and urban furniture in a way that these people can achieve at least partial independence tailored to their condition. Home care is a care providing strategy through which these patients can benefit from the capacity of the family, which requires providing standard home care requirements. Social support is one of the needs of these patients along with physical and psychological care and should be provided from the early days of the event and during the acute phase of the disease and continued at the community level for life.

Palliative care should be considered as a supportive care for SCI patients from the onset of injury until death, allowing the patients to spend their remaining time with their families while experiencing minimal pain and agony and, finally, a peaceful death. It is recommended to conduct interventional studies in future to investigate the effectiveness of the themes extracted in this study in improving the quality of life of SCI patients and their families.

## Data Availability

The datasets generated during and/or analyzed during the current study are available from the corresponding author on request.

## Abbreviations

SCI: Spinal cord injury
PAS: Physician-assisted suicide
IAHPC: International Association for Hospice and Palliative Care
